# The Sub-Classification of Amnestic Mild Cognitive Impairment Using MRI-Based Cortical Thickness Measures

**DOI:** 10.3389/fneur.2014.00076

**Published:** 2014-05-21

**Authors:** Pradeep Reddy Raamana, Wei Wen, Nicole A. Kochan, Henry Brodaty, Perminder S. Sachdev, Lei Wang, Mirza Faisal Beg

**Affiliations:** ^1^School of Engineering Science, Simon Fraser University, Burnaby, BC, Canada; ^2^Centre for Healthy Brain Ageing, School of Psychiatry, UNSW Medicine, University of New South Wales, Sydney, NSW, Australia; ^3^Neuropsychiatric Institute, Prince of Wales Hospital, Randwick, NSW, Australia; ^4^Dementia Collaborative Research Centre, School of Psychiatry, UNSW Medicine, University of New South Wales, Sydney, NSW, Australia; ^5^Feinberg School of Medicine, Northwestern University, Chicago, IL, USA

**Keywords:** amnestic, mild cognitive impairment, subtype, cortical thickness, classification, early detection, Alzheimer

## Abstract

**Background:** Amnestic mild cognitive impairment (aMCI) is considered to be the transitional stage between healthy aging and Alzheimer’s disease (AD). Moreover, aMCI individuals with additional impairment in one or more non-memory cognitive domains are at higher risk of conversion to AD. Hence accurate identification of the sub-types of aMCI would enable earlier detection of individuals progressing to AD.

**Methods:** We examine the group differences in cortical thickness between single-domain and multiple-domain sub-types of aMCI, and as well as with respect to age-matched controls in a well-balanced cohort from the Sydney Memory and Aging Study. In addition, the diagnostic value of cortical thickness in the sub-classification of aMCI as well as from normal controls using support vector machine (SVM) classifier is evaluated, using a novel cross-validation technique that can handle class-imbalance.

**Results:** This study revealed an increased, as well as a wider spread, of cortical thinning in multiple-domain aMCI compared to single-domain aMCI. The best performances of the classifier for the pairs (1) single-domain aMCI and normal controls, (2) multiple-domain aMCI and normal controls, and (3) single and multiple-domain aMCI were AUC = 0.52, 0.66, and 0.54, respectively. The accuracy of the classifier for the three pairs was just over 50% exhibiting low specificity (44–60%) and similar sensitivity (53–68%).

**Conclusion**: Analysis of group differences added evidence to the hypothesis that multiple-domain aMCI is a later stage of AD compared to single-domain aMCI. The classification results show that discrimination among single, multiple-domain sub-types of aMCI and normal controls is limited using baseline cortical thickness measures.

## 1 Introduction

There is an increased focus on developing computer-assisted tools for identifying individuals at high risk of developing Alzheimer’s disease (AD). Recent reports suggest that the amyloid pathology begins at least 20 years before any clinical symptoms appear (1–3), which highlights the importance of preclinical detection. Epidemiologic studies from across the globe have reported the annual progression rates of clinically diagnosed mild cognitive impairment (MCI) to dementia to be in the 15–25% range (4). There is also an interest in identifying sub-types of MCI, and whether these relate to specific dementia diagnoses and differential rates of conversion to dementia (5). Moreover, an association between prior subtype of MCI and subsequent progression to a particular dementia is also reported (5). The development of automated techniques for the accurate classification of MCI sub-types, hence, has important prognostic applications.

Amnestic subtype of MCI (aMCI) is found to have highest conversion rate to AD as compared to other dementias (5). There are two sub-types of aMCI based on the number of domains impaired: single-domain (sd-aMCI) and multiple-domain (md-aMCI) sub-types. There is evidence to suggest that md-aMCI is the most likely subtype to progress to AD (6) and to dementia (7, 8). Structural MRI (sMRI) is a non-invasive and economical way to capture comprehensive picture of atrophy in the brain in terms of subcortical volumetry as well as cortical thickness features. Hence it would be of value to assess the ability of structural biomarkers such as cortical thickness in accurately identifying the sub-types of aMCI.

Research in this field has so far focused on studying group differences alone, i.e., regional differences in gray matter loss or cortical thickness in pair-wise fashion. Initial attempts to study the group differences among normal controls (NC), sd-aMCI, and md-aMCI were based on voxel-based morphometry (9–11), with few studies analyzing cortical thickness (12, 13). These studies suggest that moderate differences exist. However, the sample sizes examined have been small [except for Ref. (10)] and unbalanced (9, 10, 12). In a study, where the goal is to identify which patients are at increased risk of conversion to dementia, it is important that aMCI (both single and multiple-domain sub-types) is not underrepresented. Furthermore, it is important to evaluate the diagnostic utility of these measures, which no study has previously assessed based on MRI measures (9–13). In this study, we present the first thorough assessment of classification power in cortical thickness features in identifying the sub-types of aMCI, in a well-balanced cohort.

## 2 Materials and Methods

### 2.1 Participants

The study sample was part of the Sydney Memory and Aging Study (MAS) program, which comprises community-dwelling, non-demented individuals recruited randomly through electoral roll from two electorates of East Sydney, Australia. Please refer to Ref. (7, 14) for complete details about this study. To be eligible, participants needed to be aged between 70 and 90 years old, sufficiently fluent in English to complete the psychometric assessment and were able to consent to participate. Participants were excluded if they had a previous diagnosis of dementia, psychotic symptoms or a diagnosis of schizophrenia or bipolar disorder, multiple sclerosis, motor neuron disease, developmental disability, progressive malignancy (active cancer or receiving treatment for cancer, other than prostate non-metastasized, and skin cancer), or if they had medical or psychological conditions that may have prevented them from completing assessments. Participants were excluded if they had a Mini mental Statement Examination [MMSE; (15, 16)] score of <24 adjusted for age, education, and non-English speaking background at study entry, or if they received a diagnosis of dementia after comprehensive assessment. The study was approved by the Ethics Committee of the University of New South Wales. The demographics for the current study sample are listed in Table 1.

**Table 1:** **Demographics of aMCI and normal subjects in this study**.

Diagnostic group	Total *N*	Age in years mean (SD)	Gender	Education in *N* years mean (SD)
NC	42	78.57 (4.13)	17 M + 25 F	11.97 (3.10)
sd-aMCI	38	79.92 (4.87)	25 M + 13 F	12.68 (3.53)
md-aMCI	32	78.63 (4.44)	17 M + 15 F	11.52 (3.84)

### 2.2 MAS subsample and cognitive assessments

Demographic characteristics of normal and MCI participants selected for this study from the larger MAS cohort are presented in Table 1. Participants received a comprehensive neuropsychological assessment examining the cognitive domains of memory, language, attention/processing speed, visuospatial function, and executive functions (see Table 2 for listing of test measures). Participants were classified as having MCI according to the latest international consensus diagnostic criteria and if all of the following criteria were met – a cognitive complaint from the participant or a knowledgeable informant, cognitive impairment on objective testing, they were not demented, and normal function or minimal impairment in instrumental activities of daily living. Cognitive impairment was defined as a test performance of 1.5 standard deviations (SDs) or more below published normative values (demographically adjusted where possible – Table 2). Participants were considered impaired in a domain if at least one measure in the domain was impaired. In this study, only amnestic type of MCI is included. If the impairment was restricted to the memory domain, it was classified as single-domain amnestic MCI (sd-aMCI). If an additional cognitive domain was impaired, it was classified as multiple-domain amnestic MCI (md-aMCI).

**Table 2:** **Neuropsychological tests used for MCI classifications**.

Cognitive domain	Test	Normative data source and demographic adjustments
Memory	Logical memory story A delayed recall	Education
	RAVLT	Age
	RAVLT total learning, trials 1–5	
	RAVLT short-term delayed recall; trial 6	
	RAVLT long-term delayed recall; trial 7	
	Benton visual retention test recognition	Age and education
Attention/processing speed	Digit symbol-coding	Age
	Trail making test A	Age and education
Language	Boston naming test Ñ 30 items	Age
	Semantic fluency (animals)	Age and education
Visuospatial	Block design	Age
Executive function	Controlled oral word association test (FAS)	Age and education
	Trail making test B	Age and education

*Please refer to Ref. (14) for complete details on normative data sources and related references*.

**Figure 1:**
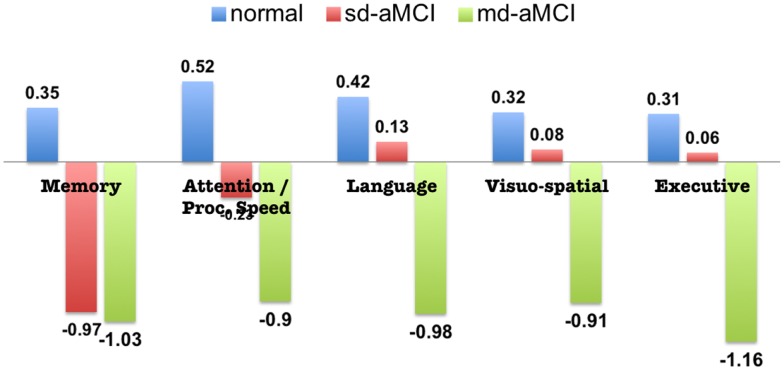
**Neuropsychological assessment of aMCI and normal subjects included in this study (standardized scores, mean)**.

Participants from non-English speaking background were excluded from the MCI groups because of the questionable validity of applying standard normative data to establish cognitive impairment in non-native English speakers (17). Of the total remaining subjects with MR imaging, subjects whose cortical parcelation did not meet our quality control, either owing to their failure in either Freesurfer cortical parcelation or estimation of cortical thickness from our Laplacian streamlines method, have been excluded. Our quality control consisted of checking for permanent failure in Freesurfer automatic parcelation, visually examining for presence of holes or handles in the pial or white surfaces (left or right hemisphere), or when the cortical surfaces have gross errors in following the structural boundaries. Further, even with acceptable Freesurfer parcelation, some subjects were excluded if our thickness computation method based on Laplacian streamlines fails to estimate thickness in either left or the right hemisphere. Within the quality controlled subset, a random subset of controls that matched in age and size with aMCI have been selected. The final selection consisted of 38 sd-aMCI, 32 md-aMCI, and 42 age-matched NC, for which the cognitive assessments are presented in Figure 1.

### 2.3 Image acquisition

The participants were scanned using a 3-T Intera Quasar scanner initially, followed by a 3-T Achieva Quasar Dual scanner, both manufactured by Philips Medical Systems, Best, The Netherlands. There was no alteration in acquisition parameters for T1-weighted sequences for both the scanners: TR = 6.39 ms, TE = 2.9 ms, flip angle = 8°, matrix size = 256 × 256, FOV = 256 × 256 × 190, and slice thickness = 1 mm with no gap between; yielding 1 × 1 × 1 mm^3^ isotropic voxels. The use of different scanners was due to reasons beyond investigator’s control and any systematic bias arising from the scanner change is unlikely given that participant recruitment was random. In fact, there were no significant differences in cortical features found between the two scanners in the Sydney MAS cohort (18). Even though there were some cohort differences across the two scanners (at age scan: scanner 1 = 77.9, scanner 2 = 79.0, *p* = 0.003; years of education: scanner 1 = 11.4, scanner 2 = 12.2, *p* = 0.013; male/female ratio: scanner 1 = 125/160, scanner 2 = 120/137, *p* = ns; the final selection of subjects in Section 2.2 are part of this larger cohort), previous studies have suggested that when vendor, field strength, and acquisition parameters remained unchanged, data collected during scanner upgrades could be pooled (19).

### 2.4 Thickness measurement and processing

Initial cortical reconstruction and volumetric segmentation of the whole brain were performed with the Freesurfer image analysis suite (20) to obtain Pial and WM/GM surfaces. The resulting cortical parcelations were quality controlled whenever possible (they were excluded otherwise). On the volume lying between these surfaces, a discrete approximation of Laplace’s equation was solved (21, 22) using the tools developed by our group. Streamlines of this harmonic function define corresponding points on the surfaces, and the Euclidean distance between these points defines the cortical thickness.

This results in thickness measurements at every vertex on the pial surface. In order to perform group-analysis, the surface of each subject in the study has been registered to the surface of a common atlas (derived from averaging over 80 healthy subjects) using the tools from Ref. (20) – see Appendix for further details. The atlas contained 327684 vertices in the whole brain. This establishes vertex-wise correspondence and enables group-wise analysis into the differences. Finally, cortical thickness was smoothed with a 10-mm full width at half height Gaussian kernel to improve the signal-to-noise ratio and statistical power for subsequent analysis (23).

### 2.5 Hippocampal features

As this study focuses on amnestic type of MCI, hippocampal features are relevant. Hence preliminary experiments on classifying the sub-types using hippocampal volumes and shape features have been performed as well (24, 25).

### 2.6 Classification using thickness features

We performed three pair-wise tests for comparison using SurfStat (26) and identified a set of regions, which are significantly different (*p* < 0.05) between each pair. The results from this group difference analysis are presented in Section 3.1. This is followed by an evaluation of accuracy of cortical thickness features in a binary classification test. The classification system consisted of intrinsic dimensionality reduction by subdividing the brain into small partitions, followed by a ranking based feature selection method and support vector machine (SVM) as classifier (27).

The dimension reduction method subdivides the cortex by partitioning each Freesurfer label (such as posterior cingulate cortex) into 10 smaller patches using the spatial clustering of vertices using *k*-means method. This results in 680 patches for the 34 cortical labels in both the hemispheres. Mean thickness value in each patch represents the feature for that partition, providing a total of 680 thickness features for each brain.

To avoid the curse of dimensionality, *T*-statistic based feature selection (top *K* features) has been performed prior to feeding the SVM classifier. For each pair, *K* is determined by the total number of samples in the corresponding binary test so as to avoid the curse of dimensionality, which is *K*_max_ = *N*/10 (28). This would give *K*_max_ = 8, 7, and 7 for the three pairs NC vs. sd-aMCI, NC vs. md-aMCI, and sd-aMCI vs. md-aMCI, respectively.

During the training phase, the parameters of the SVM classifier are tuned using grid search in the following ranges: penalty constant *C* = 10*^m^, m* = −1 to 5 and the kernel width gamma *g* = 2*^n^, n* = −5 to 4. For all the parameter combinations mentioned, the classifier is trained on a stratified training set (50% of the smallest class) and the prediction power has been evaluated on the remaining test set, and in each pair-wise classification experiment. This method is repeated 250 times, each time creating random training/test sets, in order to avoid the bias that can arise from a single training/test sets. The mean performance metrics, and their SDs, are noted. Please refer to Ref. (29) for a detailed discussion of classification method.

## 3 Results

Analysis of group differences is presented first in Section 3.1. This descriptive analysis also serves to provide regional information on significant differences among NC, sd-aMCI, and md-aMCI groups. This is followed by the evaluation of prediction power for cortical thickness using statistical learning techniques in Section 2.6.

### 3.1 Group differences

Using SurfStat (26), the differences among NC, sd-aMCI, and md-aMCI are analyzed in a pair-wise fashion and the set of vertices that are significantly different [*p* < 0.05 after correcting for multiple-comparisons using random field theory (30)] between the two groups are presented in the maps of *T*-statistic and *p*-value.

#### 3.1.1 NC vs. sd-aMCI

**Figure 2:**
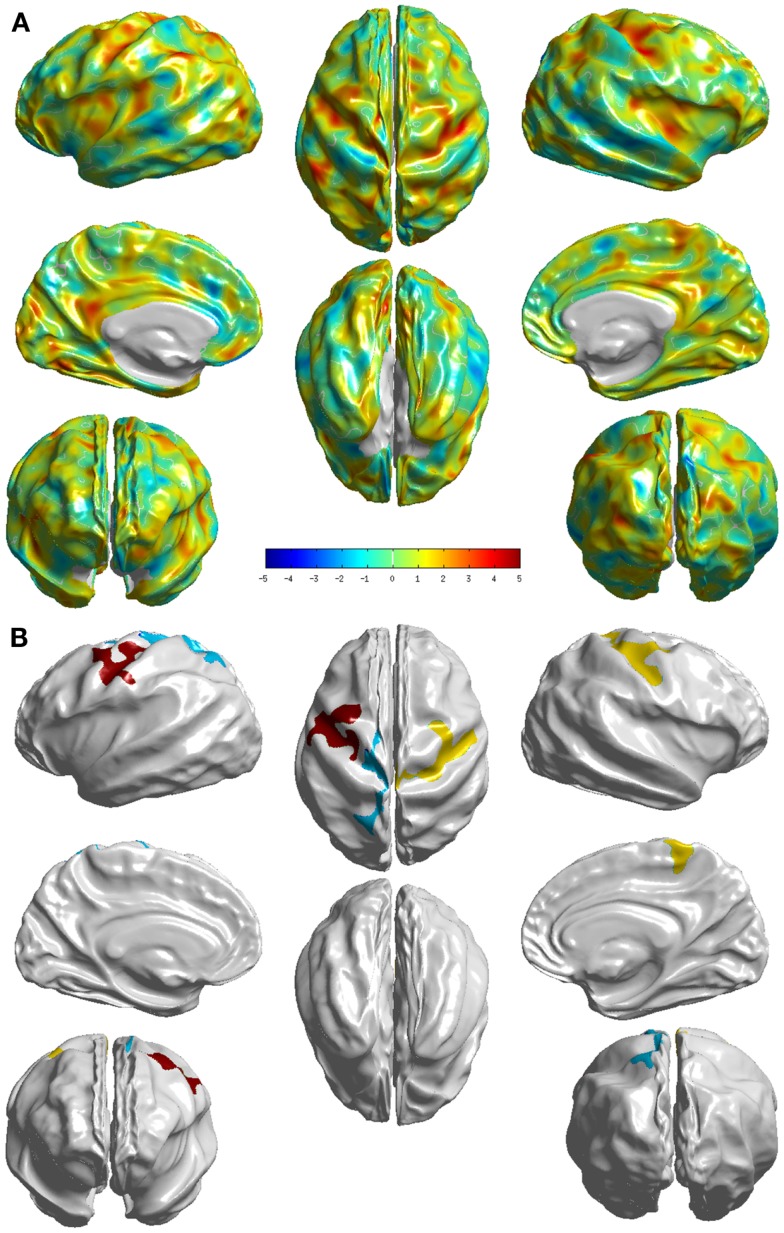
**Visualization of the differences between the two groups NC and sd-aMCI**. **(A)** *T*-statistic values displayed at each vertex **(B)** the set of clusters, which survived the multiple-comparisons test (cluster-wise significance), each colored differently. We can see that significant differences exist, although in few localized cortical areas.

The group differences between NC and sd-aMCI as measured by *T*-statistic are visualized in Figure 2A. Here, we can see that it is bright red (*T*-stat > 4) around central sulcus, meaning sd-aMCI is much thinner than NC. In fact this is the only area that survived the multiple-comparison test as visualized in Figure 2B.

#### 3.1.2 NC vs. md-aMCI

**Figure 3:**
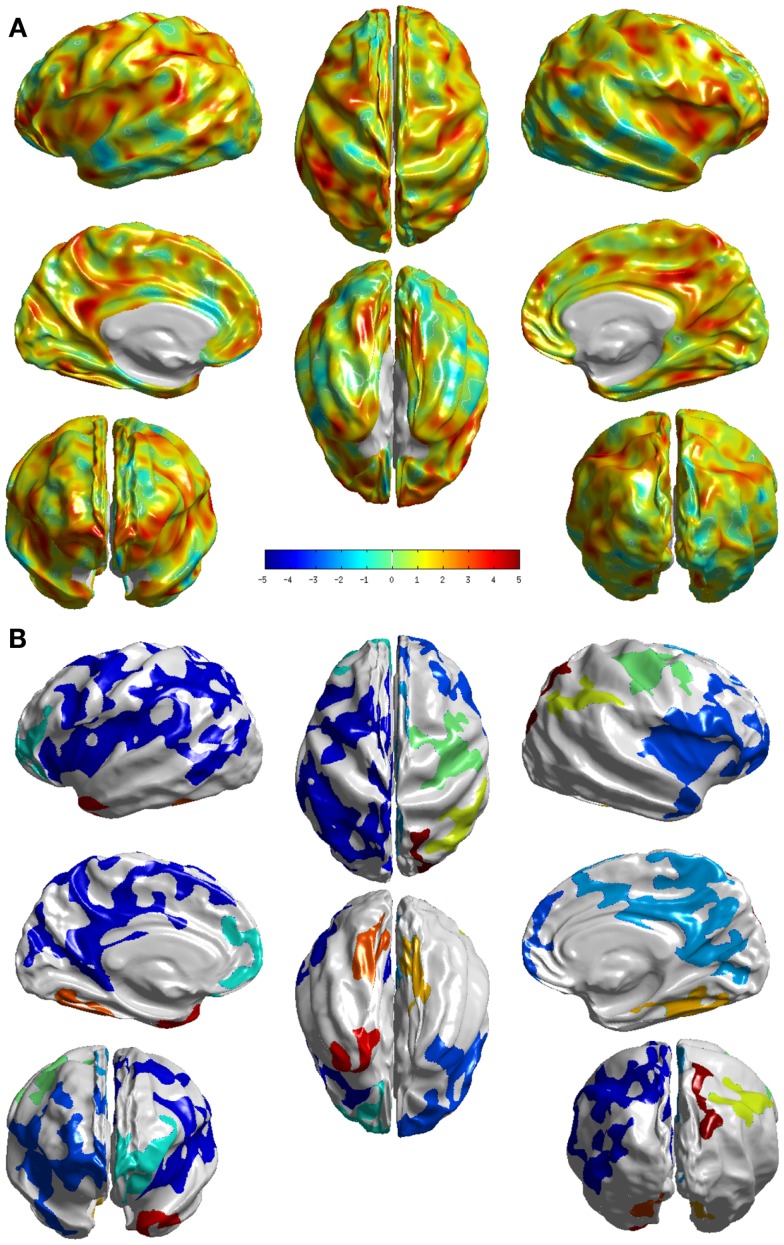
**Visualization of the differences between the two groups NC and md-aMCI**. **(A)** *T*-statistic values displayed at each vertex **(B)** the set of clusters, which survived the multiple-comparisons test (cluster-wise significance), each colored differently. We can see that they exhibit significant differences, in many cortical areas compared to the differences noticed between NC and sd-aMCI as shown in Figure 2B.

The group differences between NC and md-aMCI as measured by *T*-statistic are visualized in Figure 3A. It is immediately clear that the differences are much more widespread and thinning in md-aMCI is higher. In fact the areas (as listed in Table A2 in the Appendix) that survived the multiple-comparison test are throughout the brain as shown in Figure 3B. These are mostly complementary to the differences exhibited in NC vs. sd-aMCI, except for a slight overlap in the central sulcus.

#### 3.1.3 sd-aMCI vs. md-aMCI

**Figure 4:**
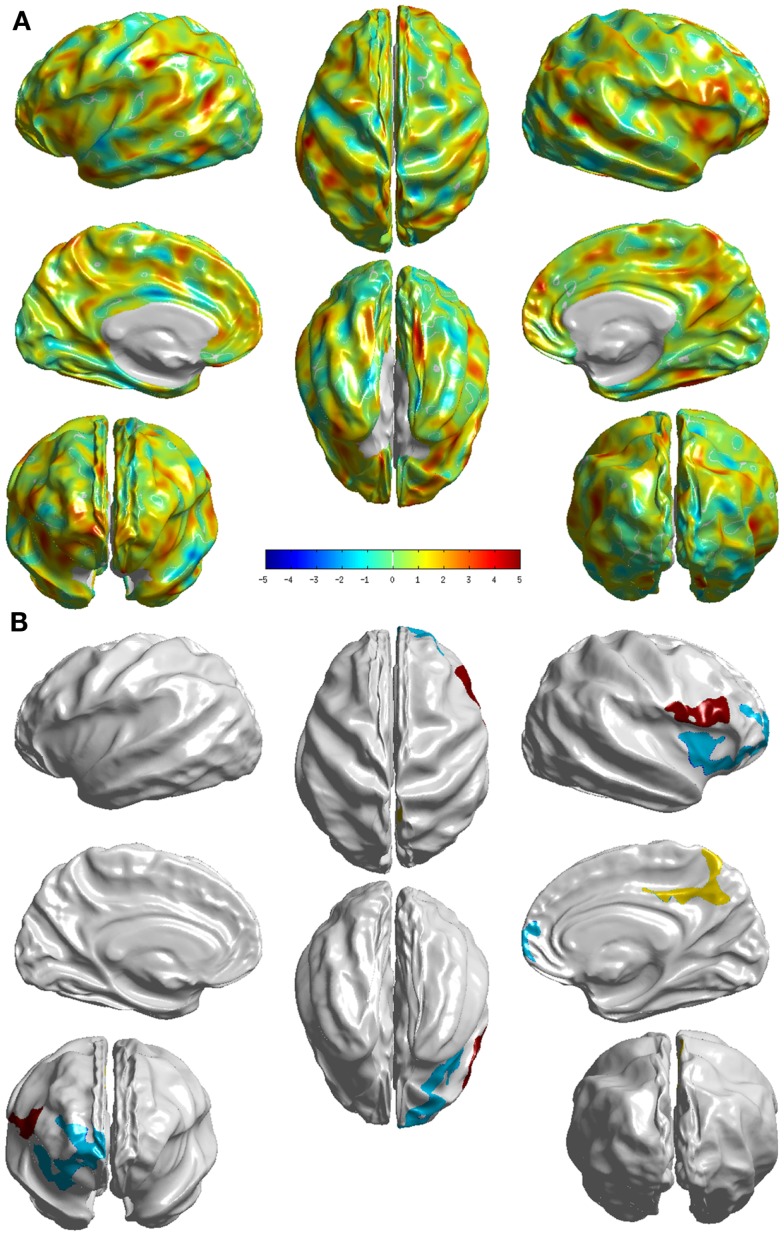
**Visualization of the differences between the two groups sd- and md-aMCI**. **(A)** *T*-statistic values displayed at each vertex **(B)** the set of clusters, which survived the multiple-comparisons test (cluster-wise significance), each colored differently. These visualizations display areas where md-aMCI is causing significantly differences as compared to sd-aMCI.

The group differences between sd-aMCI and md-aMCI as measured by *T*-statistic are visualized in Figure 4A. It can be observed that there are only few areas (as listed in Table A2 in the Appendix), which exhibit strong group differences, which can be visualized in Figure 4B (areas that survived the multiple-comparison test with *p* < 0.05). The significant differences are localized mostly in the frontal and occipital lobes.

### 3.2 Classification using hippocampal features

The classification experiments using support vector machines with hippocampal features (both left and right) revealed that hippocampal volume or shape lack any discrimination power. This is expected given that both the aMCI sub-types affect hippocampus in a similar way resulting in large overlap (see Table 3). This is consistent with findings reported in the study (24), which is based on aMCI subjects from the same MAS cohort combining both the sub-types into one group (in contrast with our study trying to discriminate the sub-types). That study assessed the power of subcortical volumetry and fractional anisotropy measures individually and in combination, to find that volumes alone didn’t have any classification power.

**Table 3:** **Volumes of hippocampi (in mm^3^) of the *a*MCI sub-types and normal controls used in this study**.

Pair	Structure	Class 1 volume in mm^3^	Class 2 volume in mm^3^	*p*-Value
NC vs. sd-aMCI	Hipp L	3437.26	3250.40	0.009*
NC vs. md-aMCI	Hipp L	3437.26	3211.33	0.001*
sd- vs. md-aMCI	Hipp L	3250.40	3211.33	0.616
NC vs. sd-aMCI	Hipp R	3359.98	3175.03	0.010*
NC vs. md-aMCI	Hipp R	3359.98	3128.40	0.005*
sd- vs. md-aMCI	Hipp R	3175.03	3128.40	0.591

*Notice the large overlap in the distribution of volumes for each structure. The results of the significance testing of whether volumes of hippocampi differ significantly between different pairs of diagnostic groups. Significant differences (*p* < 0.05) are noted with an asterisk*.

### 3.3 Classification using thickness features

**Figure 5:**
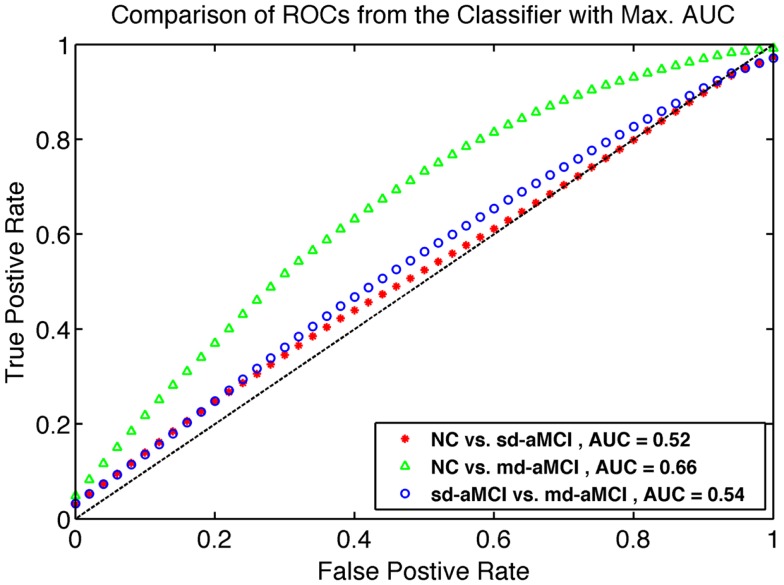
**Comparison of ROC curves for the best classifier found from grid search as described in Section 2.6**. The model from which ROC is generated are listed in Table 4.

The results for the best performance for each pair, as ranked by AUC over all the parameter sets, are shown in Table 4. The average ROCs are visualized in Figure 5, which are constructed by the vertical averaging method as described in Ref. (31), by averaging the 250 ROCs obtained from the 250 repetitions.

**Table 4:** **Comparison of the best classification performance of the classifier for each pair, and whether that performance is significantly better than random**.

Pair	Model	AUC	ACC (%)	SPEC (%)	SENS (%)	*p*-Value (AUC > Random)
NC vs. sd-aMCI	*K* = 8, *γ* = 16, *C* = 1	0.52	50	44	58	>0.05
NC vs. md-aMCI	*K* = 7, *γ* = 8, *C* = 100	0.66	61	60	62	***<*0.05**
sd-aMCI vs. md-aMCI	*K* = 7, *γ* = 4, *C* = 0.01	0.54	53	53	53	>0.05

To demonstrate that performance of the mean thickness (MT) features is significantly better than chance, additional experiments testing the *statistical significance* of the improvement in classification performance have been performed. The significance test is conducted using ROC comparison methods described in Ref. (32). The repeated cross-validation method employed in this study [known as RHsT, (29)] provides us with 250 estimates of AUC for each repetition of a cross-validation experiment. The distribution of these AUC samples for MT features are used to estimate whether it is significantly better than a random classifier (AUC of 0.5), using a non-parametric Wilcoxon rank-sum test. The result of this test is indicated in the last column of Table 4.

## 4 Discussion

We examined the group differences in cortical thickness between the two sub-types of aMCI and age-matched normal controls. Using surface-based analysis, the regions with significant differences were visualized and we have analyzed how they differed from the other pairs. We then presented an assessment of the power of cortical thickness in accurately classifying the sub-types of aMCI.

In comparison with NC, sd-aMCI presented significant differences in post central and precentral regions in both left and right hemispheres (see Figure 2). These regions are relatively robust in AD, and do not show pathology in the early stages. It *might* be possible that this is a reflection of a more generalized atrophy in the parietal and/or frontal lobes. The differences appear to cover slightly larger areas in the right hemisphere. It is interesting to note that the significant differences exist only around central sulcus and not medial temporal lobe. As the only domain of impairment in sd-aMCI is memory, we expected to see differences in the medial temporal lobe. This result is not consistent with previous findings in Ref. (9, 10, 12, 13), which reported differences in the medial temporal lobe.

In the comparison between md-aMCI and NC, the significant differences were found in the left temporal pole, left frontal pole, left superior parietal lobe, left inferior parietal lobe, left paracentral lobule, left precuneus, left posterior cingulate, left fusiform gyrus, left gyrus rectus, left superior frontal gyrus, right supramarginal gyrus, right cuneus, right temporal pole, and right lateral occipitotemporal gyrus (see Figure 3). As expected, the differences in md-aMCI (relative to NC) are much more widespread than sd-aMCI and cover a large set of regions in md-aMCI, adding evidence to the hypothesis that md-aMCI is a later stage of AD compared to sd-aMCI. Such spreading of atrophy into frontal lobe and posterior cingulate is similar to that seen in AD patients and is consistent with previous reports (10). The thinning in md-aMCI (relative to NC) covers regions functionally associated with visual perception (precuneus, cuneus, lateral occipitotemporal gyrus, and fusiform gyrus), spatial ability (parietal lobe and precuneus), language (inferior parietal, supra marginal, and frontal pole), behavioral regulation (superior frontal gyrus and frontal pole), executive function (precuneus), and motor skills (paracentral lobe). Some regions (fusiform gyrus and temporal pole) are in agreement with those reported in Ref. (9, 12), although additional differences were observed.

Relative to sd-aMCI, md-aMCI presented significantly more thinning in the right insula, right middle frontal gyrus, right precuneus, right posterior cingulate cortex, right superior frontal gyrus, right gyrus rectus, right superior frontal gyrus, and right inferior frontal gyrus (see Figure 4). This is expected as the sd-aMCI patients exhibit impairment in memory domain only and md-aMCI patients exhibit impairment in additional domains. The regions found to be significantly different are located mostly in the frontal and occipital lobes and are functionally associated with personality, behavior (frontal gyrus), attention (posterior cingulate), emotion (insula), executive, and visuospatial skills (precuneus). The differences found in posterior cingulate, temporal and frontal regions are consistent with those reported in Ref. (9, 13) and those found in precuneus are consistent with the experiments in Ref. (12). However, we find many additional differences compared to Ref. (12, 13). In our study, the differences noticed in md-aMCI relative to sd-aMCI are predominantly in the right hemisphere (see Figure 4B). Such hemispheric asymmetry to the right is consistent with Ref. (13). However, our findings are in disagreement with Ref. (12), where a left predominant atrophy is reported.

The disagreement in the set of regions found to be significantly different among the three studies may be attributed to the use of different cohorts for each study and substantial heterogeneity in the MCI construct, as well as class-imbalance in cohorts. Our cohort consists of community-dwelling residents in Sydney, Australia, whereas the cohort used in Ref. (12) comes from South Korea and the study presented in Ref. (13) is part of Alzheimer’s Disease Neuro-imaging Initiative, which sources patients from various sites in the United States. It is to be noted also that the sample sizes are unbalanced across domain types in Ref. (12), which can be another reason for detecting relatively smaller number of differences.

Analysis of the group differences and characterizing the patterns of differences is useful. Confirming the presence of differences across groups and comparing them with other studies improves our understanding of these classes. But this knowledge as such is insufficient to build an imaging biomarker that could accurately identify the different groups. Often the differences found aren’t strong enough to serve as a reliable biomarker for prediction. In this study, the classification power of cortical thickness has been assessed in accurately identifying the two sub-types of aMCI and normal controls. We performed the comparisons in a multiple pair-wise fashion using SVM as described in Section 2.6.

**Figure 6:**
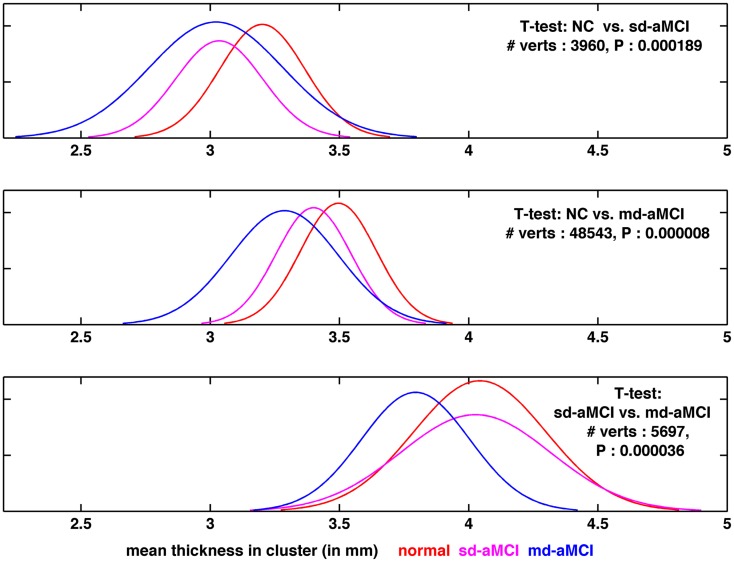
**Visualization of the distribution of thickness in the region found to be the most significantly different for each pair-wise comparison, as visualized in Figures 2B, 3B, and 4B, respectively from top to bottom**.

Looking at the best performance of the classifier for each pair, optimized via model selection and as compared in Table 4, we observe that classification performance is rather moderate. In fact, the classifier’s performance achieved significance over chance only in NC vs. md-aMCI experiment, and it was not significant in NC vs. sd-aMCI and sd-aMCI vs. md-aMCI. This is expected, as the differences among the groups are moderate at best (also see Figure 6).

We have also performed experiments in classifying the 3 groups directly in a 3-class setting with several multi-class classifiers including Decision Trees (J48) as well as multi-class SVMs. This is the first study to attempt the sub-classification of MCI, using either binary classifiers or multi-class classifiers. The best performance of the 3-class classifiers was AUC ≈ 0.6. This moderate performance is not unexpected given that the best performance of classifiers in the binary classification experiments (Table 4) is only moderate.

To gain further insight into the results, the distribution of thickness in the area found to be the most significantly different (lowest *p*-value), among those areas, which are significantly different between a given pair, has been visualized. For comparison purposes, we plotted the distribution for the remaining group as well. The comparison of thickness distribution for the three pair-wise tests is shown in Figure 6.

In the top plot of Figure 6, the histograms of mean thickness for all subjects in the most significantly different area for differences between NC (in red) and sd-aMCI (magenta) are compared. A smooth Gaussian is fitted for each histogram for ease of visualization. It is easy to see that the means of NC and sd-aMCI are separated, but there still exists a large overlap between them. The differences are enough to survive the multiple-comparison test as a cluster (Figure 2B), but not well separated. Moreover, if we compare these two groups with md-aMCI, md-aMCI completely overlaps with sd-aMCI. Very similar trends can be observed in other visualizations as well in the middle and bottom rows in Figure 6, i.e., the two groups under comparison, e.g., sd-aMCI and md-aMCI in the bottom row exhibit a small separation of means (magenta and blue curves), but still have a large overlap in the distribution. Moreover, the third group (NC) almost coincides with the group closest to it in disease severity level (in this case sd-aMCI).

Such large overlap in the thickness distribution, we believe, is the primary reason for moderate classification performance. This is expected as the differences, as seen in cortical thickness extracted from structural MRI scans, among the three groups at such an early stage of impairment are subtle at best. In addition, it is to be noted that the diagnosis of MCI is not very stable yet, e.g., high rates of reversion to normal are reported in Ref. (33, 34) and significant percentage of subjects convert to other sub-types (34). This can be another reason for moderate classification performance.

It is to be noted that one of the limitations in this study is the lack of histopathological confirmation for the clinical diagnoses employed in this study. Another limitation is the scanner upgrade midway, which is not modeled into our analysis. Even though there were minor cohort differences across the two scanners in some of demographic parameters, previous studies have suggested that when vendor, field strength, and acquisition parameters remained unchanged, data collected during scanner upgrades could be pooled (19). Another study (35) concluded that scanner upgrade did not increase the measurement variability nor introduce bias and that applying smoothing filters (which we have done with 10 mm FWHM Gaussian kernel) on the raw thickness maps can substantially reduce that thickness measurement variability. Further, the number of subjects in each diagnostic group belonging to the two scanners are: CN (scanner 1: 20 and scanner 2: 22), sd-aMCI (18/20), and md-aMCI (15/17). This shows a fairly even distribution across the two scanners, indicating that the chances of significant bias toward one scanner are greatly reduced. However, for the sake of completeness, we have performed additional experiments to investigate if there is any effect of scanner upgrade on the classification results. To this regard, we have regressed out the scanner upgrade factor from the cortical thickness features, and used the residuals to form the new set of features for classification. We repeat the classification procedure as detailed in Section 2.6, and the results (AUC of 0.52, 0.67, and 0.55 in the three pair-wise experiments, respectively) did not differ from the previous results presented in Table 4.

In conclusion, this study contributes to the important discussion of prognosis of MCI sub-types and in particular in assessing the classificatory power of sMRI features in distinguishing the sub-types of MCI. Our analysis revealed a wider spread of cortical thinning in md-aMCI (relative to NC) compared to sd-aMCI, adding evidence to the hypothesis that md-aMCI is a later stage of AD compared to sd-aMCI. Classification results from our study show that baseline cortical thickness alone does not have sufficient discriminability to differentiate normal controls, sd-aMCI, and md-aMCI from each other. However, it is currently not known whether longitudinal rates of change in thickness offer discrimination between sd-aMCI and md-aMCI, which would be worth investigating. We speculate that longitudinal features might improve the prediction accuracy of which patients are at risk of developing dementia. Fusion of subcortical features, white matter lesion features, as well as complementary features from other modalities such as FDG-PET or PiB-PET (which directly measures the presence of pathological features, if present) may substantially improve the ability in accurately identifying sub-types of aMCI.

## Conflict of Interest Statement

The authors declare that the research was conducted in the absence of any commercial or financial relationships that could be construed as a potential conflict of interest.

## Appendix

### Estimation of the common atlas

After the extraction of cortical thickness from each subject, the surface of each subject has been registered to that of a common atlas. This atlas is derived from averaging 80 healthy controls using tools from Freesurfer. With the help of Talairach transform computed for each subject, Talairach (MNI305) coordinates for each vertex are computed. These coordinates (from the 80 subjects) are averaged after mapping them to the common surface (which overlays well on the average MNI305 volume). Below, the list of all subjects that were part of this averaging are presented in Table A1.

**Table A1:** **List of IDs of the subjects used for the estimation of average atlas**.

295	1261	1280	907	981	602	484	498	681	68	1222	2	5	16	21	22	23	502	575	1035
519	520	558	883	899	1288	14	130	61	963	985	1063	403	824	843	920	1098	48	672	813
1023	1002	643	779	934	19	312	386	363	640	489	711	171	896	533	1014	173	601	405	506
605	680	260	622	863	232	969	1200	123	319	441	433	488	86	196	1194	1195	1197	1202	1203

*Note these are baseline subjects from ADNI-1*.

### Comparison of significantly differing regions across the experiments

A comprehensive comparison of the list of brain regions, which exhibited significant group differences in the three pair-wise comparisons are presented in Table A2.

**Table A2:** **Comparison of the cortical locations in the brain found to be significantly different between the three different pairs from the current study**.

sd-aMCI vs. CN	md-aMCI vs. CN	sd- vs. md-aMCI
L: precentral	On BOTH hemi	Only RIGHT
L: postcentral	Caudal anterior cingulate	Isthmus cingulate
L: superior parietal	Caudal middle frontal	Lateral orbitofrontal
L: supramarginal	Cuneus	Medial orbitofrontal
Parts of central sulcus	Fusiform	Paracentral
R: paracentral	Inferior parietal	Pars opercularis
R: postcentral	Inferior temporal	Pars orbitalis
R: precentral	Isthmuscingulate	Pars triangularis
	Lateral occipital	Postcentral
	Lateral orbitofrontal	Posterior cingulate
	Lingual	Precentral
	Medial orbitofrontal	Precuneus
	Middle temporal	Rostral middle frontal
	Parahippocampal	Superior frontal
	Paracentral	Superior parietal
	Pars opercularis	Frontal pole
	Pars orbitalis	
	Pars triangularis	
	Postcentral	
	Posterior cingulate	
	Precentral	
	Precuneus	
	Superior frontal	
	Superior parietal	
	Superior temporal	
	Supramarginal	
	Frontal pole	
	Temporal pole	
	Transverse temporal	
	Only LEFT: banks of STS	
	Entorhinal	
	Only RIGHT: pericalcarine	
	Rostral anterior cingulate	

*Please note that this is rather an exhaustive list of regions automatically generated by the program, with regions not immediately visible in the figures as they may have only few vertices part of cluster. L, Left; R, Right Hemi*.
